# Increased concentrations of conjugated bile acids are associated with osteoporosis in PSC patients

**DOI:** 10.1038/s41598-022-20351-z

**Published:** 2022-10-03

**Authors:** Julian Stürznickel, Friederike Behler-Janbeck, Anke Baranowsky, Tobias Schmidt, Dorothee Schwinge, Clara John, Ansgar W. Lohse, Christoph Schramm, Joerg Heeren, Thorsten Schinke, Michael Amling

**Affiliations:** 1 Department of Osteology and Biomechanics, University Medical Center Hamburg-Eppendorf, Lottestraße 59, 22529 Hamburg, Germany; 2 Department of Trauma and Orthopedic Surgery, University Medical Center Hamburg-Eppendorf, 20246 Hamburg, Germany; 3 Department of Biochemistry and Molecular Cell Biology, University Medical Center Hamburg-Eppendorf, 20246 Hamburg, Germany; 4 I. Department of Internal Medicine, University Medical Center Hamburg-Eppendorf, 20246 Hamburg, Germany; 5 European Reference Network on Hepatological Diseases (ERN RARE-LIVER), Hamburg Center for Translational Immunology (HCTI), University Medical Centre Hamburg-Eppendorf, 20246 Hamburg, Germany; 6 Martin Zeitz Centre for Rare Diseases, University Medical Centre Hamburg-Eppendorf, 20246 Hamburg, Germany

**Keywords:** Hepatology, Bone, Osteoporosis

## Abstract

Primary sclerosing cholangitis (PSC) is an idiopathic cholestatic liver disease characterized by chronic inflammation and progressive fibrosis of intra- and extrahepatic bile ducts. Osteoporosis is a frequent comorbidity in PSC, and we could previously demonstrate that IL17-dependent activation of bone resorption is the predominant driver of bone loss in PSC. Since we additionally observed an unexpected heterogeneity of bone mineral density in our cohort of 238 PSC patients, the present study focused on a comparative analysis of affected individuals with diagnosed osteoporosis (PSC^OPO^, n = 10) or high bone mass (PSC^HBM^, n = 7). The two groups were not distinguishable by various baseline characteristics, including liver fibrosis or serum parameters for hepatic function. In contrast, quantification of serum bile acid concentrations identified significant increases in the PSC^OPO^ group, including glycoursodeoxycholic acid (GUDCA), an exogenous bile acid administered to both patient groups. Although cell culture experiments did not support the hypothesis that an increase in circulating bile levels is a primary cause of PSC-associated osteoporosis, the remarkable differences of endogenous bile acids and GUDCA in the serum of PSC^OPO^ patients strongly suggest a yet unknown impairment of biliary metabolism and/or hepatic bile acid clearance in this patient subgroup, which is independent of liver fibrosis.

## Introduction

Primary sclerosing cholangitis (PSC) is a cholestatic liver disease with heterogenic clinical presentation and still unresolved etiology^1^ with a median age at diagnosis of 41 years, affecting men slightly more often. It is characterized by ulcerative lesions of the bile duct mucosa with chronic inflammation of intra- and extrahepatic bile ducts, causing progressive fibrosis leading to end-stage liver disease^2^. In general, PSC patients show poor response to immunosuppression^3^ and, besides liver transplantation, no curative treatment is available^1,4^. Regarding the pathogenesis, commensal bacterial communities and dysbiosis in the gut microbiome or metabolome received attention for their proposed central role in PSC^3,5^. Moreover, the altered composition of bile fluid in PSC patients was shown to favor inflammation^2^. Although there is no strong supporting data for slowing down the progression of PSC or improving survival, the majority of patients are currently treated with ursodeoxycholic acid (UDCA)^6,7^.

As recently summarized, several mechanisms of action have been proposed for the therapeutic effects of UDCA^8^, including increasing the hydrophilicity of the bile acid pool, restoring the protective bicarbonate umbrella, and an attenuation of ER stress, thereby protecting liver cells from bile acid-induced cell death. In this regard it is noteworthy that there is a high variation of endogenous bile acid concentrations and circulating levels of administered UDCA in PSC patients^9,10^. Complicating clinical management, an increased rate of liver transplantation and risk for colorectal neoplasia, among others, was observed to be associated with high UDCA dosages^11,12^.

Next to hepatic complications like portal hypertension, liver cirrhosis and associated malignancies, osteoporosis was identified to represent a major complication in a subset of PSC patients^1,13–15^. To evaluate their bone status is of central relevance, as glucocorticoid treatment among other factors is associated with further bone loss and fractures^16^. Therefore, the evaluation of bone mineral density (BMD) in PSC patients is recommended by both the American and European guidelines^6,15^. We have previously shown, in a cohort of 238 PSC patients, that the reduction of bone mass was associated with increased bone resorption correlating to a higher number of Th17^+^ cells^17^. Unexpectedly, we also observed a large heterogeneity of BMD values in this cohort. More specifically, whereas 8.2% of the patients were diagnosed with osteoporosis, some individuals displayed BMD values within or above the reference range.

In the present study we conducted a comprehensive assessment of PSC patients with either osteoporosis (PSC^OPO^, n = 10) or high bone mass (PSC^HBM^, n = 7). Revealing differences in the levels of bile acid between the two groups, we performed cell culture experiments to test the hypothesis that the systemic bile acid increase explains the low bone mass in the PSC^OPO^ group.

## Results

### PSC^OPO^ and PSC^HBM^ patients do not differ in hepatic parameters

**Figure 1:**
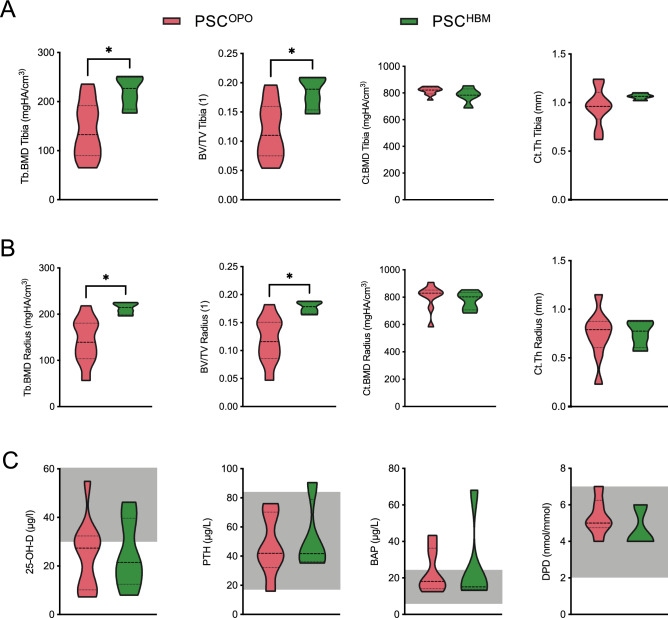
Bone microarchitecture and disease-associated parameters of PSC^OPO^ and PSC^HBM^ patients. (**A,B**) Parameters of the three-dimensional bone microarchitecture were evaluated via HR-pQCT at the distal tibia (**A**) and the distal radius (**B**). Shown is quantification of the trabecular bone mineral density (Tb.BMD), bone volume to tissue volume (BV/TV), cortical bone mineral density (Ct.BMD) and cortical thickness (Ct.Th). (**C**) Biochemical analysis was performed to evaluate relevant markers of calcium homeostasis and bone turnover, i.e., vitamin D (25-OH-D), parathyroid hormone (PTH), bone-specific alkaline phosphatase (BAP), and urinary deoxypyrodinoline (DPD). Data were analyzed by student’s t-test. *p < 0.05, **p < 0.01, ***p < 0.001, ****p < 0.0001.

We first compared baseline characteristics of PSC^OPO^ (n = 10) and PSC^HBM^ (n = 7) patients (Table 1). Interestingly, the two groups were similar regarding demographic characteristics like age or body mass index (BMI), and disease-associated parameters, such as the presence of ulcerative colitis or disease duration. Importantly, the prescribed daily dosage of UDCA did not differ significantly between both patient groups. The evaluation of bone microarchitecture by HR-pQCT reflected the findings of DXA scans and showed significantly reduced trabecular parameters for PSC^OPO^ patients at the distal tibia and radius (Fig. 1A,B). Interestingly, no significant difference was identified for cortical thickness (Ct.Th) at neither site. Biochemical analysis revealed low vitamin D levels for both groups, accompanied by PTH and bone turnover biomarker levels within the middle to upper reference range (Fig. 1C). Moreover, the assessment of biochemical markers for hepatic alterations and prognostic models in PSC did not reveal a significant difference between the two groups (Table 2).

**Table 1:** Demographic and clinical characteristics of PSC^OPO^ and PSC^HBM^ patients.

	PSC^OPO^ (n = 10)	PSC^HBM^ (n = 7)	*p*
Sex (m/f)	7/3	6/1	0.103
Age (years)	43.4 ± 12.6	44.2 ± 16.1	0.669
Height (cm)	174.4 ± 13.1	184.9 ± 10.9	0.104
Weight (kg)	70.8 ± 15.9	83.8 ± 17.5	0.130
BMI (kg/m^2^)	23.0 ± 3.2	24.3 ± 2.9	0.315
Z-score spine	−1.7 ± 1.1	1.1 ± 1.0	** < 0.0001**
Z-score hip	−2.0 ± 0.9	1.8 ± 0.6	** < 0.0001**
UDCA dose (mg/day)	1225.0 ± 700.1	1300.0 ± 404.1	0.255
Ulcerative colitis (n)	3	3	> 0.9999
Disease duration (m)	127 ± 95	56 ± 59	0.086
Leukocytes (Bill/L)	9.01 ± 7.54	9.30 ± 3.56	0.927
Lymphocytes (Bill/L)	1.97 ± 2.20	2.28 ± 0.72	0.754
IgG (g/L)	15.7 ± 7.4	12.1 ± 2.9	0.242
IgM (g/L)	1.4 ± 1.0	1.8 ± 0.6	0.417
CRP (mg/L)	11.7 ± 15.2	5.9 ± 2.3	0.338

Significant values are in bold.

**Table 2:** PSC-specific parameters in PSC^OPO^ and PSC^HBM^ patients.

	PSC^OPO^ (n = 10)	PSC^HBM^ (n = 7)	*p*
Bilirubin (mg/L)	0.88 ± 0.43	0.71 ± 0.40	0.233
AST (U/L)	41.0 ± 26.7	35.0 ± 7.5	0.790
ALP (U/L)	166.2 ± 113.7	190.3 ± 151.2	0.918
Fibroscan (kPa)	8.7 ± 5.8	8.5 ± 3.6	0.931
Amsterdam-Oxford model score	1.3 ± 0.4	1.7 ± 0.3	0.098
Mayo risk score	−0.18 ± 0.85	−0.20 ± 0.61	0.955
MELD score	7.2 ± 0.8	6.9 ± 0.9	0.415

### Increased concentrations of conjugated bile acids in PSC^OPO^ patients

**Figure 2:**
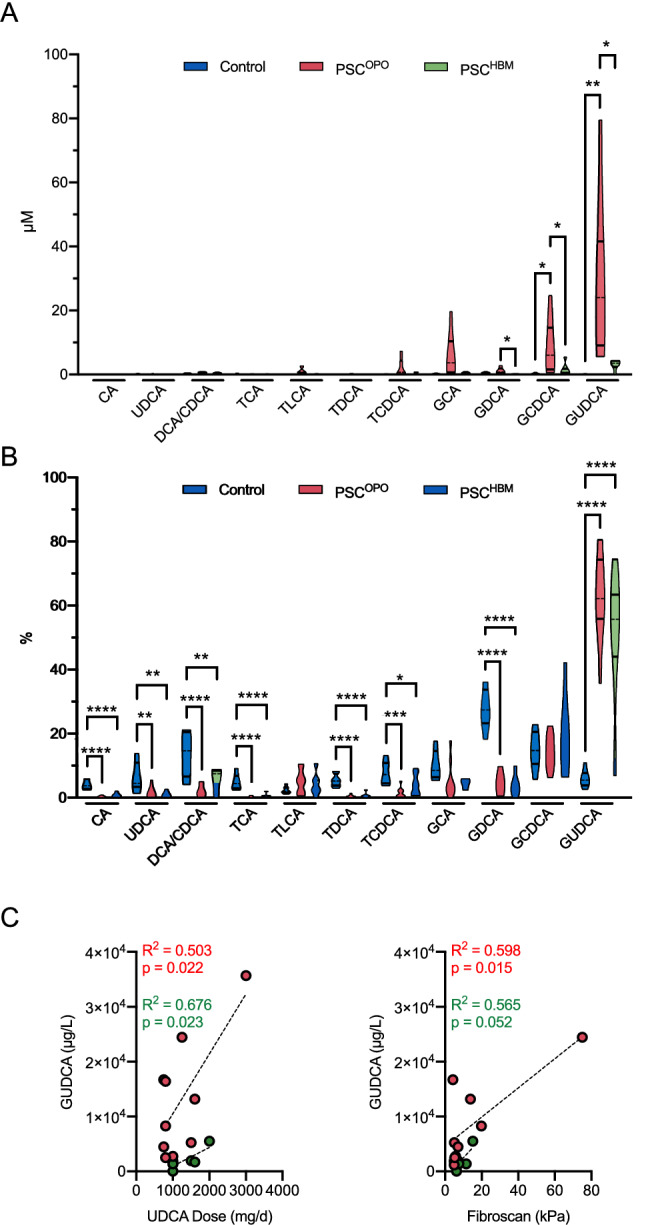
Bile acid concentrations in the serum of PSC^OPO^ and PSC^HBM^ patients. (**A**) Concentrations of unconjungated and conjugated bile acids were measured in the serum of control individuals and of PSC^OPO^ and PSC^HBM^ patients. (**B**) Shown is the percentage of the indicated bile acids when related to the total bile acid pool. (**C**) Linear regression analyses were performed to investigate the association of UDCA dosage with GUDCA levels (left) and of Fibroscan values with GUDCA levels (right). Data were analyzed by one way ANOVA with Tukey’s multiple comparison test and linear regression model. *p < 0.05, **p < 0.01, ***p < 0.001, ****p < 0.0001.

We next measured the serum concentrations of unconjugated and conjugated bile acids in the two patient groups and compared them to sera from age- and sex-matched healthy donors. Surprisingly, a significant increase in the levels of glycine-conjugated bile acids was detected only in the PSC^OPO^ group (Fig. 2A). More specifically, levels of glycodeoxycholic acid (GDCA), glycochenodeoxycholic acid (GCDCA), and glycoursodeoxycholic acid (GUDCA) were significantly elevated in PSC^OPO^ compared to PSC^HBM^ patients. Remarkably, however, the composition of the bile acid pool was equally altered in PSC^OPO^ and PSC^HBM^ patients, i.e. more than 50% of the total bile acids represented GUDCA (Fig. 2B). Linear regression analysis revealed significant associations with GUDCA levels and the daily prescribed UDCA dosage for both groups (Fig. 2C). Importantly, however, the slope differed between PSC^OPO^ and PSC^HBM^ to a large extent, indicative for higher levels of GUDCA in PSC^OPO^ despite equivalent UDCA supplementation compared to PSC^HBM^. The same applied to FibroScan measurements, revealing significantly higher GUDCA levels in PSC^OPO^ patients despite comparable liver stiffness parameters in both PSC groups. Overall, these findings are indicative for an impaired bile acid metabolism and/or clearance in PSC^OPO^ patients.

### Bile acids do not impair osteogenic differentiation of mesenchymal progenitor cells

**Figure 3:**
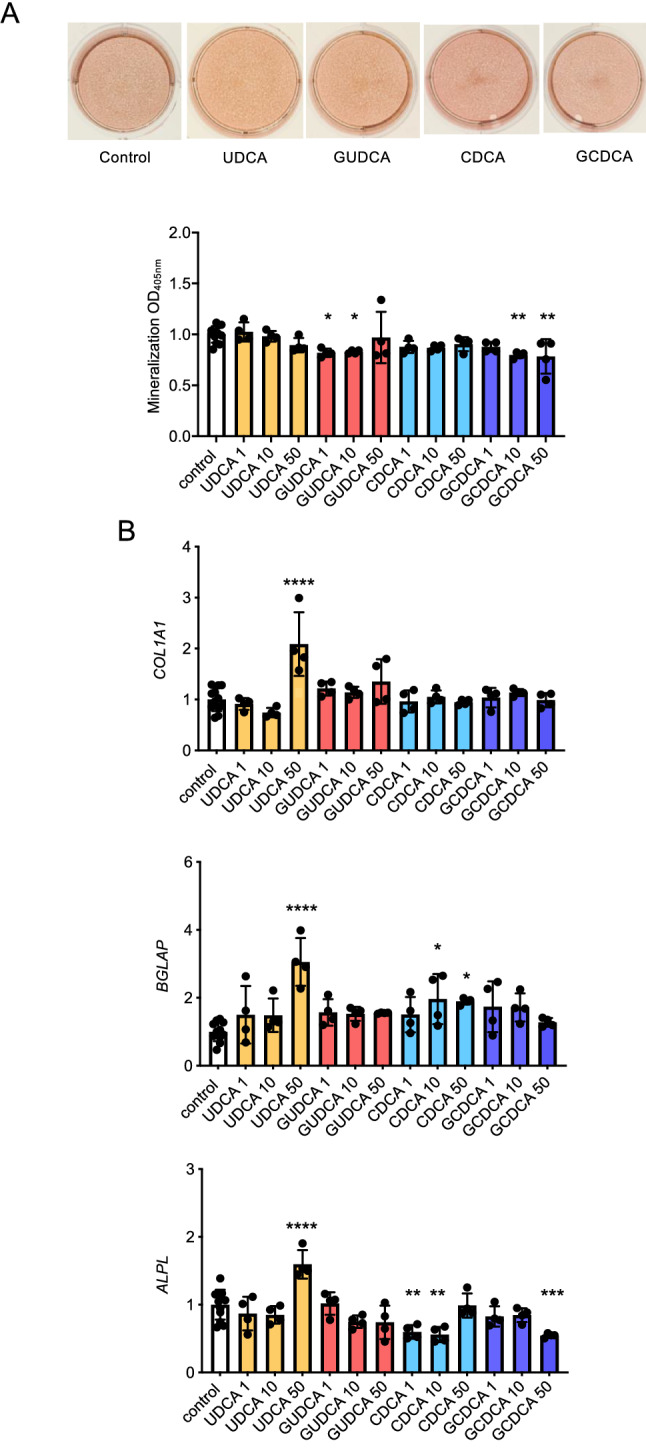
Influence of bile acids on osteogenic differentiation of hMSC-TERT cells. (**A**) Representative images after alizarin red staining of hMSC-TERT cultures differentiated for 10 days in the presence of unconjugated (UDCA, CDCA) or conjugated (GUDCA, GCDCA) bile acids at concentrations of 1 µM, 10 µM or 50 µM, as indicated. Quantification of matrix mineralization is shown below. (**B**) qRT-PCR expression analysis of the osteogenesis markers *COL1A1, BGLAP,* and *ALPL* after day 10 of differentiation. *p < 0.05, **p < 0.01, ***p < 0.001, ****p < 0.0001.

We next addressed the question if increased levels of circulating bile acids would potentially cause the low bone mass pathology in PSC^OPO^ patients. For that purpose, we first assessed the influence of GUDCA and GCDCA, the two most strongly increased bile acids in PSC^OPO^ patients, as well as their unconjugated forms, on osteoblast differentiation. We therefore took advantage of the immortalized mesenchymal cell line hMSC-TERT, where osteogenic differentiation was induced for 10 days. Extracellular matrix mineralization was only moderately affected by bile acid supplementation, but there was no concentration dependency observed (Fig. 3A). We further analyzed the expression of three relevant osteoblast differentiation markers, i.e., *COL1A1, BGLAP* and *ALPL* (Fig. 3B). Whereas we did not observe a dose-dependent reduction in the expression of these genes, there was a significant positive influence of 50 µM UDCA on the expression of all three osteogenesis markers. However, since the serum levels of unconjugated UDCA were much lower in the PSC^OPO^ patients, these observations are unlikely to cause the observed PSC-associated bone pathologies.

### GUDCA differentially affects expression of *CLNC7* and *CTSK* in human osteoclasts

**Figure 4:**
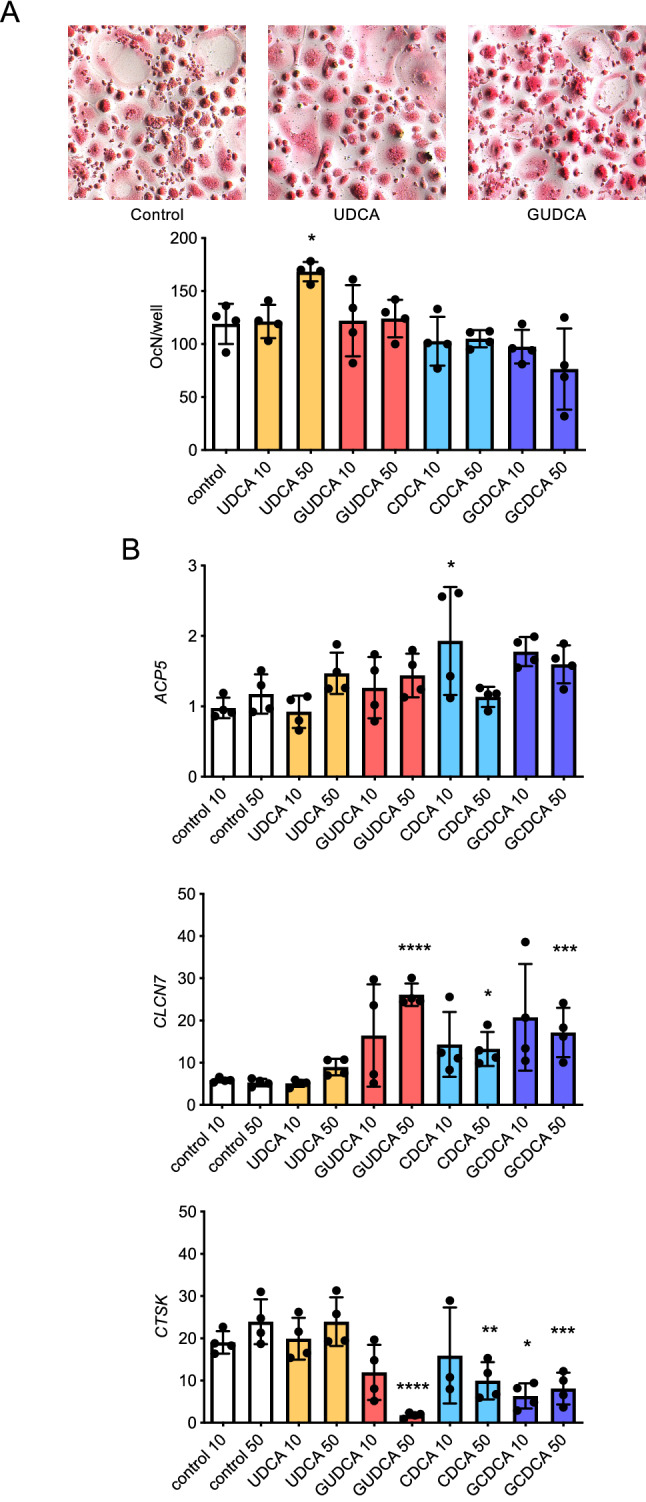
Influence of bile acids on osteoclastogenesis of peripheral blood mononuclear cells. (**A**) Representative images after TRAP activity staining of peripheral blood mononuclear cells differentiated for 14 days in the presence of M-CSF and RANKL. Quantification of multinucleated osteoclasts generated in the absence or presence of the indicated bile acids, at concentrations of 10 µM or 50 µM, is shown below. (**B**) qRT-PCR expression analysis of the osteoclast markers *ACP5*, *CLCN7*, and *CTSK* after 14 days of differentiation. *p < 0.05, **p < 0.01, ***p < 0.001, ****p < 0.0001.

To analyze the impact of the same set of bile acids on osteoclastogenesis, we took advantage of mononuclear cells isolated from buffy coats, which were differentiated for 14 days in the presence of M-CSF and sRANKL^18^. After staining the cultures for TRAP activity, we detected a significantly increased number of osteoclasts in the presence of 50 µM UDCA (Fig. 4A), which we do not consider as clinically relevant for the same reasons as outlined above. By monitoring the expression of three relevant osteoclastogenesis markers, i.e. *ACP5, CLCN7* and *CTSK* (Fig. 4B), we unexpectedly observed a specific influence of GUDCA at a concentration of 50 µM. In fact, while *CLCN7* expression was more than fourfold increased towards control cultures, *CTSK* expression was reduced by more than 80% in the presence of GUDCA. A similar influence was also observed with 50 µM GCDCA, albeit to a lower extent. Taken together, these results suggest that GUDCA, at a concentration range found in the serum of PSC^OPO^ patients, can affect expression of specific genes in osteoclasts.

## Discussion

In the present study we performed a comparative characterization of PSC patients with either osteoporosis or high bone mass. The three-dimensional skeletal analysis by HR-pQCT indicated differences primarily in the trabecular bone compartment, whereas cortical bone characteristics were comparable between PSC^OPO^ and PSC^HBM^ patients*.* Remarkably, the two groups showed similar demographic and disease-associated characteristics, and laboratory markers were not able to explain the differences in bone mass. Likely due to supplementation, the bile acid pool in both PSC groups consisted to more than 50% of GUDCA. However, there were striking differences between PSC^OPO^ and PSC^HBM^ patients in terms of bile acid concentrations, which were most pronounced for GCDCA and GUDCA. Importantly, the increased levels of endogenous and exogenous bile acids in the serum of the PSC^OPO^ patients were not explained by a higher degree of detectable liver pathologies. This observation is in full agreement with our previous analyses of 238 PSC patients, where we did not observe a correlation between BMD and disease duration of liver fibrosis^17^.

One obvious question based on our findings was if the increased levels of circulating bile acids would affect bone remodeling and potentially cause the decreased trabecular bone mass in PSC^OPO^ patients. Indeed, there are previous studies suggesting that bile acids may influence the activity of bone remodeling cell types^19,20^, and an influence of the farnesoid X receptor (FXR), which functions as an intracellular bile acid sensor, on bone formation and resorption has been described^21,22^. Importantly, however, it has not been addressed yet, if the conjugated forms of bile acids would cause similar influences, which is a key question, since they are known to act through different receptor signaling complexes when compared to the unconjugated forms^23^.

We therefore assessed the impact of the two most strongly elevated bile acids in the serum of PSC^OPO^ patients, in their unconjugated and conjugated forms, on osteoblast and osteoclast differentiation. While the influences of UDCA, GUDCA, CDCA or GCDCA on osteogenic differentiation of the human mesenchymal cell line hMSC-TERT were subtle, there was an unexpected specificity in the transcriptional response of human osteoclasts towards 50 µM GUDCA. Since the mean serum concentration of GUDCA in the PSC^OPO^ group was 25 µM, with two patients exceeding the value of 50 µM, these cell culture data are potentially relevant. Importantly, however, since the main influence of GUDCA was a repression of *CTSK* expression, it is unlikely that this effect would cause the PSC^OPO^ pathology, since CTSK inactivation or blockade is known to increase bone mass^24^. In conclusion, there is no evidence supporting a contribution of elevated GUCDA or GCDCA serum concentrations to the osteoporosis in a subset of PSC patients. However, although the differences observed for other bile acids (GDCA, GCA, TCDCA or TLCA) between PSC^OPO^ and PSC^HBM^ patients were much less pronounced, we cannot rule out that these subtle changes may contribute to bone loss in the PSC^OPO^ group. On the other hand, since our previous studies with a cohort of 238 PSC patients strongly suggested that IL17-dependent activation of bone resorption is the major driver of bone loss in PSC patients, which was subsequently confirmed in mouse models^17^, we hypothesize that increased bile acid concentrations in the PSC^OPO^ group are not directly causative for their low BMD.

Some limitations of this study need to be mentioned. First, the number of individuals is comparably small. This is primarily explained by our focus on a subset of patients from the previously analyzed cohort^17^, where we essentially compared the two extremes with respect to BMD. It is certainly required to confirm the findings of increased bile acid concentrations in osteoporotic PSC patients with a validation cohort. However, since only 17 of the 238 patients fulfilled the criteria for the present study, this requires a substantial number of newly identified patients. Second, no microbiome, metabolome, or transcriptome analysis were performed in these patients. Since 9 out of 18 PSC patients (including one of the PSC^HBM^ group) displayed a more than 100-fold increase of serum GUDCA levels compared to controls, we truly believe that such comprehensive studies should be considered in the future. Especially since the vast majority of PSC patients receive UDCA supplementation, it is potentially important to identify and to understand, why specific patients, regardless of their bone status, show such a strong GUDCA accumulation in the serum. It might also be informative to systematically monitor serum GUDCA levels in PSC patients, including patients with normal BMD or osteopenia, and to correlate these data with microbiome and/or transcriptome studies to clarify the interplay between microbial dysbiosis, proinflammatory signaling, bile acid metabolism and associated bone mass regulation.

## Materials and methods

### Patient cohort

From a cohort of 238 PSC patients, we included 17 individuals (10 PSC^OPO^ and 7 PSC^HBM^) for this study, in whom within close temporal context (6 months maximum) skeletal status, hepatic parameters and bile acid concentrations were analyzed. To evaluate liver stiffness, FibroScan measurements were performed as previously reported^17^. All individuals were admitted to the Department of Osteology and Biomechanics, after diagnosis of PSC was established by the First Department of Medicine of the University Medical Center Hamburg-Eppendorf, Germany. The subgroup allocation was based on BMD measurement (i.e., PSC^OPO^: aBMD T-score ≤ -2.5, PSC^HBM^: aBMD T-score ≥ 1.0), and all patients receiving bone-specific treatment (e.g., denosumab) or prednisolone were excluded from the analyses. This study was performed in accordance with the Declaration of Helsinki and approved by the local ethics committee of the University Medical Center Hamburg-Eppendorf (Ethikkomission Ärztekammer Hamburg, PV4081-Z). Written informed consent was obtained from all included patients and healthy donors.

### Skeletal assessment

The assessment included areal bone mineral density (aBMD) measurement by dual-energy X-ray absorptiometry (DXA; Lunar iDXA, GE Healthcare, Madison, WI, USA), which was performed at the lumbar spine (L1-4) and both proximal femora. The values are presented as T-scores (standard deviation to sex-matched individuals 20–29 years of age from the NHANES III database) and Z-scores (standard deviation to healthy, sex-matched individuals of the same decade). In addition to aBMD evaluation, three-dimensional bone microarchitecture analysis by high-resolution peripheral quantitative computed tomography (HR-pQCT, XtremeCT, Scanco Medical, Brüttisellen, Switzerland) was performed according to the default settings (60 kVp, 9000 µA, 100 ms integration time, and 82 µm voxel size) and the standardized in vivo protocol as described previously by our group^25^. HR-pQCT measurements were carried out at both the non-dominant distal radius and the contralateral distal tibia. The scans were evaluated with protocols provided by the manufacturer and the nomenclature follows the *American Society of Bone and Mineral Research*^26^.

### Biochemical analysis

Serum and urine samples were obtained and analyzed at the local laboratory. These serum markers included 25-hydroxyvitamin D (25-OH-D), parathyroid hormone (PTH), bone-specific alkaline phosphatase (BAP), bilirubin, aspartate aminotransferase (AST), and alanine aminotransferase (ALT). Additionally, urinary levels of deoxypyridinoline (DPD) were measured to evaluate bone resorption.

### Quantification of bile acids

We performed targeted bile acid and hydroxysterol analysis by high-performance liquid chromatography (HPLC) coupled to electrospray ionization tandem mass spectrometry as recently described^27^. In brief, sample preparation comprised a simple methanol liquid–liquid extraction of tissues and biofluids. Quantitative measurement of bile acids was performed using a HPLC-ESI-QqQ system in multiple reaction monitoring (MRM) mode [HPLC: 1200 Infinity Quaternary LC System (Agilent); column: Accucore Polar Premium (2.6 μm, 150 mm × 2.1 mm inner diameter, Thermo Fisher Scienctific, Inc.); QqQ: API 4000 Q trap (ABSCIEX)]. Peak identification and quantification were performed by comparing retention times, as well as MRM transitions and peak areas, respectively, to corresponding standard chromatograms. For bile acid quantification we also included sera from age- and sex-matched healthy donors (n = 8).

### Osteoblast differentiation

To analyze the influence of non-conjugated and conjugated bile acids on osteoblast differentiation, we used the cell line hMSC-TERT^28,29^. Human MSC-TERT cells (subclone 20) were seeded at a density of 3 × 10^4^ cells/well on a 12-well plate and cultured in DMEM, high glucose GlutaMAX (Gibco, Life Technologies, USA) supplemented with 10% fetal calf serum (FCS, Gibco Life Technologies, USA), 5% l-glutamine (200 mM, Gibco, Life Technologies, USA) and 1% penicillin/streptomycin (10,000U/ml, Gibco, Life Technologies, USA). At 80% confluence, designated day 0, osteoblast differentiation was induced by enrichment of the culture medium with 50 µg/ml ascorbic acid (Sigma-Aldrich, USA) and 10 mM β-glycerophosphate (Sigma-Aldrich, USA). At the same time, the cells were treated with different concentrations of either conjugated or unconjugated bile acids. UDCA was added, either in its non-conjugated form (UCDA, U5127, Sigma Aldrich, USA) or conjugated to glycine (GUDCA, 06863, Sigma Aldrich, USA) at a concentration of 1, 10 or 50 µM during the whole course of differentiation. The same concentrations were added for chenodeoxycholic acid (CDCA, 10011286, Cayman Chemical, USA) and its glycine-conjugated form (GCDCA, 16942, Cayman Chemical, USA). All bile acids were solved in dimethyl sulfoxide (DMSO) that acted also as a control. Osteogenic medium supplemented with bile acids was changed every third day. At day 10 of differentiation and treatment, cells were either harvested in TRIzol for RNA extraction or stained with alizarin red to quantify the mineralization of the cells. Alizarin red staining and quantification of mineralization was performed as described previously^30^.

### Osteoclast differentiation

Primary human mononuclear cells were isolated from buffy coats from healthy donors and were provided by the Department for Transfusion Medicine, Hamburg University School of Medicine, Germany. Density gradient centrifugation with Ficoll Paque Plus was used to separate the mononuclear osteoclast precursor cells from other formed elements in blood. To remove contaminating lymphocytes, the cells were purified for adherence. The non-adherent cells were washed off with RPMI 1640 at day 1 of culture, so that only the adherent monocytes were cultivated. For the generation of osteoclasts α-MEM (Sigma-Aldrich) was used, supplemented with 15% FBS (v/v) (Life Technologies), 100U/ml penicillin/streptomycin (Life Technologies), M-CSF (20 ng/ml) and sRANKL (40 ng/ml). UDCA, GUDCA, CDCA or GCDCA were added to the medium at concentrations of 10 mM and 50 µM. After 14 days of differentiation, osteoclasts were stained for activity of tartrate-resistant acid phosphatase (TRAP) according to standard protocols (23). At the same time point, cultures with identical treatment were used for RNA extraction as described above.

### Gene expression analysis

For gene expression analysis, RNA was isolated using peqGOLD TriFast™, according to manufacturer’s instructions. Concentration and quality of RNA were measured using a NanoDrop ND-1000 system. Complementary DNA synthesis was performed using the Verso cDNA Synthesis Kit. Expression analysis by qRT-PCR was performed using a StepOnePlus system and predesigned TaqMan gene expression assays (*ALPL*: Hs00758162_m1*, BGLAP:* Hs01587813_g1, *COL1A1*: Hs00164004_m1, *ACP5*: Hs00356261_m1*, CLCN7:* Hs01126462_m1 and *CTSK:* Hs00166156_m1). The presented data are given as relative expression towards internal controls, i.e. *RPLP0* or *GAPDH* for osteoblast and osteoclast differentiation, respectively.

### Statistical analysis

Statistical analysis was performed using Prism 8.4.0 software (GraphPad Software, Inc., La Jolla, CA, USA). The results are given as absolute values or the mean standard deviation (SD). Data were analyzed by Grubbs’ test and significant outliers (α = 0.05) were excluded from further analysis. The normal distribution of the data was tested by the Shapiro–Wilk test. Student’s t test was performed for unpaired data of two groups; otherwise, the paired t-test was used for paired data. For comparison of three or more groups, one-way ANOVA and repeated measures with Tukey correction was performed. To investigate association between selected factors, linear regression models were applied. The level of significance was defined as p < 0.05.

## Data Availability

All data are available in the main text.
